# Prevalence of malaria resistance-associated mutations in *Plasmodium falciparum* circulating in 2017–2018, Bo, Sierra Leone

**DOI:** 10.3389/fmicb.2022.1059695

**Published:** 2022-12-02

**Authors:** Tomasz A. Leski, Chris Rowe Taitt, Sophie M. Colston, Umaru Bangura, Andrew Holtz, Chadwick Y. Yasuda, Nathanael D. Reynolds, Joseph Lahai, Joseph M. Lamin, Victoria Baio, Rashid Ansumana, David A. Stenger, Gary J. Vora

**Affiliations:** ^1^Center for Bio/Molecular Science and Engineering, U.S. Naval Research Laboratory, Washington, DC, United States; ^2^Mercy Hospital Research Laboratory, Bo, Sierra Leone; ^3^College of Science, George Mason University, Fairfax, VA, United States; ^4^Njala University, Freetown, Sierra Leone

**Keywords:** *Plasmodium falciparum*, drug resistance, antimalarials, Sierra Leone, artemeter, lumefantrine, antifolates

## Abstract

**Introduction:**

In spite of promising medical, sociological, and engineering strategies and interventions to reduce the burden of disease, malaria remains a source of significant morbidity and mortality, especially among children in sub-Saharan Africa. In particular, progress in the development and administration of chemotherapeutic agents is threatened by evolved resistance to most of the antimalarials currently in use, including artemisinins.

**Methods:**

This study analyzed the prevalence of mutations associated with antimalarial resistance in *Plasmodium falciparum* from 95 clinical samples collected from individuals with clinically confirmed malaria at a hospital in Bo, Sierra Leone between May 2017 and December 2018. The combination of polymerase chain reaction amplification and subsequent high throughput DNA sequencing was used to determine the presence of resistance-associated mutations in five *P. falciparum* genes – *pfcrt, pfmdr1, pfdhfr, pfdhps* and *pfkelch13*. The geographic origin of parasites was assigned using mitochondrial sequences.

**Results:**

Relevant mutations were detected in the *pfcrt* (22%), *pfmdr1* (>58%), *pfdhfr* (100%) and *pfdhps* (>80%) genes while no resistance-associated mutations were found in the *pfkelch13* gene. The mitochondrial barcodes were consistent with a West African parasite origin with one exception indicating an isolate imported from East Africa.

**Discussion:**

Detection of the *pfmdr1* NFSND haplotype in 50% of the samples indicated the increasing prevalence of strains with elevated tolerance to artemeter + lumefantrine (AL) threatening the combination currently used to treat uncomplicated malaria in Sierra Leone. The frequency of mutations linked to resistance to antifolates suggests widespread resistance to the drug combination used for intermittent preventive treatment during pregnancy.

## Introduction

Significant progress has been made in the global struggle against malaria over the last two decades, culminating with the recent recommendation of RTS,S/AS01 as a vaccine for children under 5 years of age in sub-Saharan Africa (World Health Organization, 2021b). Malaria case incidence, defined as cases per 1,000 population at risk, decreased from 81 in the year 2000 to 59 in 2020 with 10 countries certified by the World Health Organization (WHO) as malaria-free and several more achieving three consecutive years of zero indigenous malaria cases (World Health Organization, 2021c). More significantly, global malaria mortality rates (deaths per 100,000 population at risk) dropped by half – from 30 to 15 in the same period (World Health Organization, 2021c). These decreases were the direct consequence of both non-pharmaceutical interventions such as the use of insecticide-treated mosquito nets and vector control efforts, as well as improvements in malaria treatments and diagnostics. Development and worldwide deployment of artemisinin-based combination therapies (ACTs) for treatment of uncomplicated malaria caused by *Plasmodium falciparum* are widely credited for the sharp decrease of malaria mortality rates. ACTs are currently recommended by WHO as the first line of therapy for *P. falciparum* infections in malaria-endemic countries (World Health Organization, 2021c), replacing mono- and combination therapies using antimalarial compounds to which extensive resistance has developed globally (Haldar et al., 2018; Conrad and Rosenthal, 2019; World Health Organization, 2021c). While resistance to artemisinin was first documented in western Cambodia in 2008 (Noedl et al., 2008) and is currently common in Southeast Asia (Noedl et al., 2008; Dondorp et al., 2009; Ashley et al., 2014), ACTs have remained highly effective on the African continent. However, the emergence of strains carrying artemisinin resistance-associated mutations have been reported from multiple locations in Africa (Conrad and Rosenthal, 2019; Uwimana et al., 2020; Arya et al., 2021) and local spread of clinically resistant strains was also documented recently (Balikagala et al., 2021). Emergence and spread of artemisinin resistance has the potential to undo much of the progress achieved since the beginning of the 21st century. Therefore, monitoring of the underlying mechanisms of antimalarial resistance in this part of the world is now increasingly necessary.

Widespread surveillance of antimalarial resistance in low- and middle-income countries is complicated by the high financial and logistical costs associated with therapeutic efficacy studies [TES; the current gold standard used to determine antimalarial drug efficacy (World Health Organization, 2010, 2021c)] and *in vitro* studies. On the other hand, genomic mutations associated with resistance are well-known for a number of antimalarial therapeutics, making detection and tracking of drug resistant strains much simpler (Haldar et al., 2018; Conrad and Rosenthal, 2019). The most extensive data on antimalarial resistance markers are available for a handful of *P. falciparum* genes: mutations in the *pfcrt* and *pfmdr1* genes are associated with resistance to chloroquine (CQ), other 4-aminoquinoline drugs, piperaquine (PPQ) and arylamino alcohols (e.g., quinine, QN) (Foote et al., 1990; Fidock et al., 2000; Ross et al., 2018); mutations in the *pfdhps* and *pfdhfr* genes are associated with antifolate resistance (Triglia et al., 1997; Basco and Ringwald, 2000); and mutations in the propeller and BTB/POZ domains of the *pfkelch13* gene are associated with artemisinin resistance (Ariey et al., 2014; Paloque et al., 2021). Mutations or copy number changes of several other *P. falciparum* genes such as *pfpm2* (encoding plsmepsin 2) (Amato et al., 2017), *pfcytb* (encoding cytochrome B) (Korsinczky et al., 2000), *pfmdr6* (Gendrot et al., 2017), *pfmrp1* (Dahlstrom et al., 2009), *pfnhe1* (Bennett et al., 2007), *pftetQ* and *pfmdt* (Briolant et al., 2010) as well as mutations in apicoplast genes (Blasco et al., 2017) are also associated with resistance to antimalarials.

Sierra Leone remains a moderate to high transmission country for malaria, reporting over 725 thousand cases in 2020 (World Health Organization, 2021c). Mortality due to malaria has decreased significantly since 2000 in large part due to public availability of free diagnostic testing, widespread deployment of insecticide-treated bed nets, and national implementation of intermittent preventive treatment in both infants (IPTi) and pregnant women (IPTp) (World Health Organization, 2021c). However, malaria remains the leading cause of death in Sierra Leone (Carshon-Marsh et al., 2022). Furthermore, the potential for rising resistance to antimalarials is a significant concern, as data on the spread and mechanisms of resistance within West Africa, specifically in Sierra Leone and its neighbors, are limited. For example, the Worldwide Antimalarial Resistance Network (WARN) database records between years 2000 and 2017 contain only five clinical efficacy studies of artemisinin combinations in Guinea, Sierra Leone and Liberia with only one study in Sierra Leone. Data from other West African countries, while sparse, are somewhat less limited (Worldwide Antimalarial Resistance Network, 2021). According to WARN, the studies of prevalence of molecular markers of resistance to antimalarials in Sierra Leone and its neighbors are similarly scarce.

In this study, 95 samples collected from patients in Bo, Sierra Leone in 2017 and 2018 with polymerase chain reaction (PCR)-confirmed *P. falciparum* infection were analyzed for the presence of resistance-associated mutations in five genes (*pfcrt, pfmdr1, pfdhps, pfdhfr* and *pfkelch13*) to determine the prevalence and types of mutations.

## Materials and methods

### Sample collection

Whole blood samples were obtained by venipuncture as part of a larger study that sought to analyze the causes of febrile illness and validate infectious disease diagnostics in Bo, Sierra Leone. This protocol was approved by the Sierra Leone Ministry of Health and Sanitation Ethics and Scientific Review Committee and human subjects research Institutional Review Boards of the US Naval Research Laboratory (NRL.2012.0007) and George Mason University (477605). Informed consent from patients (or, for minors under the age of 18, consent from their parents) was obtained and documented prior to collection of biological specimens or clinical data. Samples were collected from 95 individuals with PCR-confirmed *P. falciparum* malaria between 16 May 2017 and 6 December 2018. Each sample was assigned a specific alphanumeric code before it was stripped of any identifiers that could be traced back to an individual subject.

### Deoxyribonucleic acid extraction

Deoxyribonucleic acid (DNA) was extracted from 200 μl venous blood using the QIAamp DNA Mini Kit (Qiagen, Germantown, MD, USA) according to the manufacturer’s instructions and resuspended in a final volume of 200 μl.

### Resistance marker analysis

A modified version of a previously published method (Nag et al., 2017) was used to analyze antimalarial resistance-associated polymorphisms in five nuclear genes (*pfcrt, pfmdr1, pfdhfr, pfdhps* and *pfkelch13*) and parasites’ origins using “mitochondrial barcodes” (Preston et al., 2014). This approach used an initial PCR to amplify 12 gene fragments encompassing the polymorphisms in the five nuclear genes and three additional mitochondrial gene fragments. The primers used in this first PCR reaction included (universal) end-sequences complementary to indexing primers. A second PCR was conducted to add barcodes unique to each sample and sequencing adapters for follow-on Illumina MiSeq library preparation and sequencing. Barcoded amplicons for all individual samples were pooled before sequencing.

#### Fragment amplification

A previously published primer set was initially used for both amplifications (Nag et al., 2017). The primer pair used to amplify of *pfmdr1* gene fragment closest to the 5’ end of the gene (Pfmdr1.1-F/R) was redesigned to amplify a longer fragment including sequences corresponding to codon 184, another resistance-associated polymorphism (and renamed to Pfmdr1.1a-F/R). See Supplementary Table 1 for all primer sequences used for the first round of PCR amplifications. The published amplification strategy using five multiplexed and two singleplex amplifications was initially followed (Nag et al., 2017) (see Supplementary material for details). However, subsequent sequencing failed to produce sequences of adequate quality for some of the amplicons. These missing fragments were amplified individually under re-optimized conditions (see Supplementary material), and a second sequencing library was generated to fill in gaps in the sequencing data.

All amplifications were run on 1.2% agarose FlashGels (Lonza, Rockland, ME, USA) to verify amplicon sizes and quantified fluorometrically using a Qubit 3.0 fluorometer with the dsDNA Broad Range reagent kit (Thermo Fisher Grand Island, NY, USA).

#### Amplicon indexing

Amplicons from each sample were combined into a pool such that the final concentration of each amplicon was 0.7 ng/μl (assuming that multiplexed reactions produced equal amounts of all amplicons). The pooled preparations were used as templates for indexing amplifications using previously published indexing primers (Nag et al., 2017). A unique combination of forward and reverse indexing primers allowed amplicons from each sample to be identified (Supplementary Table 2). The indexing reactions were performed essentially as previously described except that the annealing temperature was set to 60°C and the number of amplification cycles was increased to 32. After size confirmation (1.2% agarose FlashGels), indexed amplicons were quantified using the Qubit 3.0/dsDNA Broad Range reagent kit.

#### Amplicon pooling and purification

For the initial sequencing [with amplicons obtained according to the original method (Nag et al., 2017)], the indexed amplicons were combined into eight pools containing 5 ng/μl of indexed amplicons from each sample with 11–12 samples represented in each pool. For the second sequencing library (containing amplified fragments from re-optimized singleplex PCRs), indexed amplicons were combined into four separate pools with amplicons representing 22–30 samples in each pool; volumes of the indexed amplicons in the pools were adjusted to obtain 0.5 ng/μl for each indexed amplicon to ensure their equimolar representation. Pooled amplicons were purified, quantified and combined as described in the original method (Nag et al., 2017). Two separate libraries were created in this way, the first one containing amplicons generated according to the original method (Nag et al., 2017) and the second one for missing amplicons not covered in the first library and amplified individually.

#### Sequencing

The amplicon library processing and sequencing using a MiSeq benchtop sequencer (Illumina, San Diego, CA, USA) was conducted as described in the original method (Nag et al., 2017). A custom sample sheet was used to demultiplex the samples. Run metrics were monitored using the Sequencing Analysis Viewer.

#### Sequence data analysis

The general quality of raw sequencing reads was assessed using FastQC v.0.11.9. A custom workflow was programmed in CLC Genomics Workbench v.11.0.1 (Qiagen) to trim sequences based on quality (phred score = 20, ambiguous limit = 2, 3’ base removal = 1) and to remove adapter, primer and PhiX sequences. Processed reads were mapped to reference sequences using the following parameters: match score = 1, mismatch score = 1, insertion cost = 3, deletion cost = 3, length fraction = 0.75, similarity fraction = 0.85, random mapping. Sequence variants were determined from the mapping results using basic variant detection 2.0 with thresholds for minimum count = 5, coverage = 100, central quality = 30, neighborhood quality = 20. Consensus sequences were extracted at a threshold of 100 and a noise threshold of 0.75.

#### Alignments

For each gene, the obtained consensus sequences were aligned with each other and with reference sequences using the web version of MAFFT software and web interface hosted by EMBL-EBI (Madeira et al., 2019). The following GenBank reference sequences from *P. falciparum* strain 3D7 were used in this study: *pfcrt* – XM_001348968.1, *pfmdr*1 – XM_001351751.1, *pfdhfr* – XM_001351443.1, *pfdhps* – XM_001349382.1, *pfkelch13* – XM_001350122.1, complete mitochondrial genome – CP017005.1.

## Results

### Prevalence of single nucleotide polymorphisms associated with antimalarial resistance

MiSeq Run 1 generated a total of 20,480,006 filter passed reads with 15,410,960 reads mapped to reference sequences. Run 2 generated a total of 14,567,396 filter passed reads with 11,054,917 reads mapped to reference sequences.

Table 1 shows a summary of the prevalence of all tested SNPs associated with resistance. Note that this table combines the data generated under conditions described by Nag et al. (2017) and subsequent sequencing using fragments amplified individually under optimized conditions.

**TABLE 1:** Prevalence of amino acid substitutions in analyzed samples.

Gene	Amino acid position^1^	Amino acid substitution	Prevalence of substitution (%)	Prevalence of mixed samples (%)^2^
*pfcrt*	**72**	C → S	0	16
	**73**	V → deletion	0	
	**74**	M → I	22	
	**75**	N → E	22	
	**76**	K → T	22	
*pfmdr*1	34	K → R	1	56
	**86**	N → Y	7	
	111	I → L	1	
	**184**	Y → F	58	
	**1034**	S → C	0	
	**1042**	N → D	0	
	1069	T (synonymous)	6	
	1191	M → I	1	
	1204	S (synonymous)	2	
	**1246**	D → Y	4	
*pfdhfr*	**16**	A → V	0	0
	**51**	N → I	100	
	**59**	C → R	100	
	**108**	S → N	100	
	**164**	I → L	0	
*pfdhps*	**431**	V → I	0	24
	**436**	S → A	29	
	**437**	A → G	80	
	**540**	K → E	7	
	**581**	A → G	0	
	**613**	A → S	8	
*pfkelch*13	33	K (synonymous)	1	36
	177	L → I	1	
	185	N → K	1	
	189	K → T	40	
	189	K → N	5	
	207	T (synonymous)	1	
	**446**	F → I	0	
	**458**	N → Y	0	
	**476**	M → I	0	
	**493**	Y → H	0	
	493	Y (synonymous)	1	
	**539**	R → T	0	
	**543**	I → T	0	
	**553**	P → L	0	
	**561**	R → H	0	
	**574**	P → L	0	
	**580**	C → Y	0	
	707	Q (synonymous)	1	

^1^Numbers in bold font denote well known antimalarial resistance-associated polymorphisms.

^2^Mixed samples are defined as the samples in which sequencing shows >20% of an alternative base for SNPs. All samples with at least one SNP with evidence of mixed infection were counted to calculate the percentage of mixed samples for a particular gene.

#### Pfcrt

Mutations in the *pfcrt* gene covered by the sequences analyzed here have been associated with changes in the sensitivity of *P. falciparum* to CQ, other 4-aminoquinolines, and arylamino alcohol antimalarials (Haldar et al., 2018; Conrad and Rosenthal, 2019). Sequences of adequate quality were obtained for 65% of the *pfcrt* amplicons; therefore, we were not able to determine the presence of single nucleotide polymorphisms for 35% of the samples tested. For the obtained sequences, SNPs resulting in amino acid substitutions were found at positions 74, 75, and 76 and no SNPs were detected at positions 72 and 73 (CVIET haplotype, see Table 2 for haplotype frequencies). The prevalence of the triple mutant (CVIET haplotype) was found in 22% of all successfully sequenced samples.

**TABLE 2:** Haplotype rates.

Gene	Haplotype^1^	Description^2^	Haplotypes determined (*n*)	Prevalence (%)
*pfcrt*	CVMNK	Wild type	63	78
	CV**IET**	Triple mutant (74 + 75 + 76)		22
*pfmdr1*	NYSND	Wild type	66	42
	N**F**SND	Single mutant (184)		50
	**Y**YSN**Y**	Double mutant (86 + 1246)		5
	**YF**SND	Double mutant (86 + 184)		3
*pfdhfr*	ANCSI	Wild type	95	0
	A**IRN**I	Triple mutant (51 + 59 + 108)		100
*pfdhfs*	SAKAA	Wild type	83	10
	S**G**KAA	Single mutant (437)		55
	**AG**KAA	Double mutant (436 + 437)		11
	**A**AKAA	Single mutant (436)		11
	**AG**KA**S**	Triple mutant (436 + 437 + 613)		6
	S**GE**AA	Double mutant (437 + 540)		7

^1^Amino acid residues in bold font are resistance-associated substitutions.

^2^Numbers in parentheses indicate the positions of amino acids which were changed compared to the wild type.

#### Pfmdr1

Mutations in the *pfmdr1* gene have been shown to have a modulating effect on CQ resistance and are associated with resistance to arylamino alcohols (QN, mefloquine (MQ), and others) (Haldar et al., 2018; Conrad and Rosenthal, 2019). The sequencing success rate for *pfmdr1* amplicons ranged from 81 to 95%. SNPs resulting in substitutions at protein positions 86, 184, and 1,246 were observed with 7, 58, and 4% prevalence, respectively. No SNPs corresponding to amino acid positions 1,034 or 1,042 were found. Out of 66 samples for which the full haplotype could be determined, 28 (42%) were wild type for all five loci (NYSND haplotype), 33 (50%) were single mutants (codon 184, NFSND haplotype), three (5%) had SNPs corresponding to substitutions at codon positions 86 and 1,246 (YYSNY haplotype), and two (3%) had polymorphisms corresponding to substitutions at codon positions 86 and 184 (YFSND haplotype; Table 2). Additional non-synonymous SNPs were found in single samples corresponding to amino acid positions 111 and 1,191. Synonymous SNPs corresponding to the amino acid positions 1,069 and 1,204 were also detected in 6% and 2% of the sequenced samples, respectively.

#### Pfdhfr and pfdhps

Mutations in the *pfdhfr* and *pfdhps* genes are associated with resistance to folate pathway antagonists including pyrimethamine (PYR, *pfdhfr*) and sulfadoxine (*pfdhps*) which are currently used exclusively as a drug combination (Haldar et al., 2018; Conrad and Rosenthal, 2019). All *pfdhfr* gene amplicons were sequenced successfully, and 90–94% of the *pfdhps* gene amplicons yielded good quality sequences. SNPs in *pfdhfr* sequences resulting in amino acid substitutions at positions 51, 59 and 108 were found in all samples while no SNPs were detected at locations corresponding to codon positions 16 and 164. The *pfdhfr* haplotype determined for all samples was therefore AIRNI (with wild-type codon corresponding to A at position 16, three mutations corresponding to the following substitutions: N51I, C59R, S108N and the wild-type codon corresponding to I at position 164, see Table 2). SNPs in the *pfdhps* sequences resulting in amino acid substitutions at positions 436, 437, 540, and 613 were found with 20, 11, 7, and 8% prevalence, respectively. No SNPs corresponding to amino acid 431 were observed. Out of 83 samples for which the full haplotype could be determined, eight (10%) were wild type (SAKAA haplotype), 55 (66%) harbored a single amino acid substitution at codon 437 (SGKAA haplotype) or codon 436 (AAKAA haplotype), 16 (18%) were double mutants with SNPs corresponding to substitutions at codon positions 436 and 437, and five (6%) had three amino acid changes at codons 436, 437 and 613 (AGKAS haplotype; see Table 2).

#### Pfkelch13

Mutations in the propeller domains of the *pfkelch13* gene [the region of the encoded protein between amino acids 416 and 692 (Conrad and Rosenthal, 2019)] are responsible for artemisinin resistance in Southeast Asia (Ariey et al., 2014) and have been reported in several locations in Africa, though the specific SNPs observed in Africa have not been definitively linked to resistance (Conrad and Rosenthal, 2019; Arya et al., 2021). The five amplicons sequenced for *pfkelch13* covered the entire length of the gene and their sequencing success rate ranged from 84 to 100% depending on the amplicon. Non-synonymous SNPs resulting in amino acid substitutions were found at positions corresponding to codon 177 (one sample), 185 (one sample) and 189 (45% of all sequenced samples) – all outside the propeller domains. There were two different SNPs observed affecting the 189 amino acid position: one resulting in a K → T substitution (40% of sequenced samples) and the other resulting in a K → N substitution (5% of sequenced samples). Non-synonymous SNPs were not found in the resistance-associated propeller domains of *pfkelch13*. Several synonymous SNPs were found in individual samples in codons corresponding to amino acids 33, 207, 493 and 707.

### Mitochondrial barcodes

Base identity in five polymorphic positions in three mitochondrial genes associated with geographic origin could be determined in 93 of the 95 samples; for the remaining two samples only one or two base identifications were made. Of these, the barcodes (CTTAG) were consistent with a West African origin of *Plasmodium* parasites in 92 samples. The barcode of one sample (MEDx144, collected in 2018), contained A at position 2,383, indicating an East African origin.

### Prevalence of mixed samples

Reads from both sequencing runs were searched for evidence of mixed infections. Samples were identified as mixed when more than 20% reads for a particular polymorphic position contained an alternative base. The prevalence of mixed samples ranged from 6% when looking at mitochondrial sequences up to 56% based on the *pfmdr1* gene fragment sequences (Table 1). The overall rate of mixed sequences, when combining results from all analyzed gene fragments, was 79%.

## Discussion

While limited in size, the current study was designed to give baseline prevalence numbers for various genetic markers documented to be associated with lowered susceptibility or resistance to several major classes of antimalarials: the 4-aminoquinolines (CQ, amodiaquine (AQ) and PPQ), folate pathway inhibitors (sulfa drugs, PYR, proguanil), arylamino alcohols (QN, lumefantrine (LUM), and MQ), and artemisinin compounds. For this reason, we assessed the presence/absence of 29 drug resistance-associated SNPs in five *P. falciparum* genes using a combination of PCR and high-throughput sequencing.

Introduced in the 1950s, CQ has historically been the most widely used antimalarial in Africa but was discontinued in the early 2000s due to widespread resistance (Flegg et al., 2013). A related 4-aminoquinolone drug, AQ, is now used in a number of ACTs for treatment of uncomplicated malaria. Cross-resistance to AQ in CQ-resistant strains has been widely documented in many geographic regions, but has been less problematic in Africa, likely due to different mechanisms, historic selective pressure, and genetics (Sa et al., 2009). Here we observed a number of SNPs located in the *pfcrt* and *pfmdr1* genes that were associated with CQ and AQ resistance. Specifically, *pfcrt* SNPs responsible for amino acid substitutions at positions 74, 75 and 76 (M74I, N75E and K76T) were observed in 22% of the studied samples; all three mutations were always present together with no substitutions at positions 72 and 73 (CVIET haplotype). The K76T mutation is highly predictive for CQ and AQ resistance and 100% prevalence has been cited by several studies in East Africa; prevalence of K76T throughout West Africa has been estimated to range from 20 to 80% (Kublin et al., 2003; Frosch et al., 2011; Veiga et al., 2016; Zhou et al., 2016; Lu et al., 2017; Zhang et al., 2018; Conrad and Rosenthal, 2019). No observations of the AQ-resistance-conferring SVMNT haplotype (C72S + K76T) were made; the SVMNT haplotype has been documented in other parts of Africa and is associated with AQ selective pressure (Mehlotra et al., 2008; Sa et al., 2009; Gama et al., 2010). The moderate prevalence of CVIET and absence of SVMNT haplotypes in the analyzed population suggest that CQ or a similar antimalarial may still be used in Sierra Leone despite official public health policy of exclusive use of ACTs [artesunate plus AQ (AS + AQ) or artemeter plus LUM (AL)] for treatment of uncomplicated malaria.

SNPs in the *pfmdr1* gene also have a modulating effect on parasite susceptibility to several ACT partner drugs (e.g., LUM, MQ, AQ, and PPQ) and CQ, potentially by increasing the fitness of strains with *pfcrt* SNPs resulting in the K76T substitution (Roepe, 2009). In this study, the SNP in *pfmdr1* resulting in the N86Y substitution was found in seven samples, none of which harbored the *pfcrt* CVIET haplotype sequences. N86Y confers a decrease in CQ susceptibility (but not resistance) when present with wild-type *pfcrt*, but significantly increases CQ and AQ resistance when found in isolates with *pfcrt* CVIET haplotypes (Veiga et al., 2016). The SNP in the *pfmdr1* gene resulting in the Y184F substitution was observed in more than half (56%) of the analyzed samples, but the overall prevalence of haplotypes based on codons 87 and 184 of *pfmdr1* differed significantly from a previous study of over 1,000 West African samples (Veiga et al., 2016). The SNPs corresponding to C-terminal D1246Y substitutions were observed in four samples, which is consistent with other studies showing a low prevalence of this SNP in African isolates (Mbaye et al., 2016; Veiga et al., 2016; Achol et al., 2019). Interestingly, the prevalence of the *pfmdr1* single mutant haplotype NFSND (also referred to as 86 + 184 + 1,246 haplotype NFD) was 50% among all samples for which the haplotype could be determined. This haplotype develops when faced with selective pressure from AL (artemeter plus LUM) administration (Malmberg et al., 2013b; Thomsen et al., 2013; Jovel et al., 2015) and has been linked to a 15-fold higher *in vivo* AL tolerance (Malmberg et al., 2013a). This finding suggests that use of AL for treatment of confirmed, uncomplicated malaria in Sierra Leone is causing the selection of less sensitive *P. falciparum* strains.

Sulfadoxine-pyrimethamine (SP) is another widely used antimalarial and is the principal drug combination used for IPTi and IPTp in Sierra Leone and other African countries (World Health Organization, 2021c). Resistance to each partner drug in this anti-folate combination is conferred by point mutations in genes encoding two key enzymes in the folate biosynthetic pathway, dihydropteroate synthase (*pfdhps*) and dihydrofolate reductase (*pfdhfr*), respectively. In this study, *pfdhfr* SNPs corresponding to substitutions N51I, C59R and S108N were found in all analyzed samples. This set of mutations confers high level resistance to PYR. At the same time, 80% of these samples also harbored the *pfdhps* SNP resulting in substitution A437G; this change causes a 5-fold increase of resistance to sulfadoxine (Triglia et al., 1998). Additional SNPs in *pfdhps* resulting in substitutions that increase the level of sulfadoxine resistance were found at lower prevalence – S436A (26%), K540E (7%), and A613S (8%). The K540E substitution is considered a marker of high sulfadoxine resistance; when present in combination with three *pfdhfr* mutations (N51I, C59R, S198N) and A437G in *pfdhps* (quintuple mutant), K540E is highly associated with SP failure as a therapeutic and has reduced efficacy for IPTp (Omar et al., 2001; Kublin et al., 2002; Braun et al., 2015). Though the quintuple mutant is prevalent in the eastern part of the continent, to date, it has rarely been observed in West Africa (Bwijo et al., 2003; Okell et al., 2017; Kayode et al., 2021). Its presence in 7% of the tested samples may be a harbinger of rising levels of SP resistance within the local population and portend movement away from this drug combination, especially if the WHO decides to modify its existing policies related to malaria chemoprevention (World Health Organization, 2021a).

The high potency and limited half-life of artemisinin and its derivatives have spurred the use of ACTs to allow clearance of remaining parasites via action of the longer-lived partner drugs. For this reason, ACTs are now used as first-line therapeutics for uncomplicated malaria worldwide, including Sierra Leone (World Health Organization, 2021c). Development of resistance to artemisinin, whether developed independently or via importation from southeast Asia, would likely lead to failure of ACTs and a resultant public health crisis throughout all of Africa (Conrad and Rosenthal, 2019). For this reason, it is critically important to monitor the artemisinin resistance-linked mutations in the propeller and BTB/POZ domains of the *pfkelch13* gene (KPBD) in sub-Saharan Africa. The current study found no SNPs resulting in non-synonymous substitutions within these domains, which is in line with several recent studies in the region (Zhou et al., 2016; Nag et al., 2017, 2019; Idowu et al., 2019). Non-synonymous mutations different from those observed in Southeast Asia have been detected in Africa but none have been unequivocally linked to high resistance and therapeutic failure (Menard et al., 2016; Arya et al., 2021). On the other hand, a large number of SNPs resulting in amino acid substitutions were observed in the gene region corresponding to the N-terminal domain of the K13 protein not associated with artemisinin resistance. Specifically, SNPs corresponding to K189T/N substitutions were present in 45% of sequenced samples. This substitution has previously been reported as relatively prevalent within this region, but has not been linked to artemisinin resistance (Nag et al., 2017; Idowu et al., 2019).

This study had several limitations. The study size was relatively small (*n* = 95 subjects) and complicated by sequencing failures in one or more markers for 53% of the tested population. Therefore, the data described may not be representative of Bo District or Sierra Leone in general. Furthermore, the dearth of historic information on marker prevalence within Sierra Leone restricts our ability to assess changes of their prevalence over time. For the analysis of the *pfcrt* gene, the primers used in this study were the same as the in the original method (Nag et al., 2017) and as such did not encompass the sequences corresponding to protein positions downstream from the 72–76 region. Amino acid substitutions located in several codon positions ranging from codon 93–353 in PfCRT were shown to be responsible for increased resistance to PPQ found in *P. falciparum* isolates form Cambodia (Ross et al., 2018) and China (Small-Saunders et al., 2022). Modified primers should be used in the future studies to explore this important resistance mechanism to PPQ which is used as partner drug in DHA + PPQ combination therapy. Given the study’s emphasis on sequence aspects of antimalarial resistance, neither copy number variations (i.e., gene amplification) that may contribute to resistance to MQ, other arylamino alcohols and DHA (e.g., *pfmdr1* amplification) (Cowman et al., 1994; Cui et al., 2012) nor gene deletions (e.g., genes coding for PfEMP1) that were shown to affect artemisinin resistance *in vitro* (Lee et al., 2021) were considered. Finally, other genes not *directly* associated with resistance may indirectly affect antimicrobial resistance by increasing fitness where the presence of various SNPs has a fitness cost [e.g., *pfgch1* and PYR (Nair et al., 2008; Kumpornsin et al., 2014)]; these effects were not within the scope of this study.

In summary, the prevalence of resistance SNPs explored in this study was generally consistent with other studies conducted recently in this region (Zhou et al., 2016; Lu et al., 2017; Nag et al., 2017, 2019; Zhang et al., 2018; Idowu et al., 2019). We found no evidence of artemisinin resistance-associated mutations in *pfkelch13* but did observe moderate levels of *pfcrt* mutations responsible for CQ resistance, most likely due to the continued use of CQ or related antimalarials. We further observed *pfmdr1* gene alleles (NFSND haplotype) responsible for decreased sensitivity to LUM, the partner drug in one of the key ACT regimens for the treatment of uncomplicated malaria used in Sierra Leone. High levels of antifolate resistance-associated mutations were found in the *pfdhfr* and *pfdhps* genes suggesting high resistance to the SP drug combination currently used for intermittent preventive treatment during pregnancy. While not addressing gene amplification and other factors indirectly associated with antimalarial resistance, this study provides much needed baseline data on the prevalence of genetic resistance markers. The generation of additional baseline data is encouraged, as Sierra Leone currently has no national program for the surveillance of antimalarial resistance that can be used to inform public health policies.

## Data availability statement

The datasets presented in this study can be found in online repositories. The names of the repository/repositories and accession number(s) can be found in the article/Supplementary material.

## Ethics statement

This protocol was approved by the Sierra Leone Ministry of Health and Sanitation Ethics and Scientific Review Committee and human subjects research Institutional Review Boards of the US Naval Research Laboratory (NRL.2012.0007) and George Mason University (477605). Written informed consent to participate in this study was provided by the participants’ legal guardian/next of kin.

## Author contributions

TL conceived the study and designed the experimental protocols. TL and CT developed the human subject protocol. CT, UB, AH, CY, NR, JL, JML, VB, and RA performed or oversaw the sample collection and nucleic acid extraction in Sierra Leone. TL, CT, SC, AH, and CY performed the laboratory sample processing, amplification, sequencing and data analysis. TL and CT wrote the first version of the manuscript. SC, RA, DS, and GV reviewed and made edits to the manuscript. DS and GV obtained the funding for this work. All authors read and approved the submitted version of the manuscript.
