# Supplementary material for: Prevalence of malaria resistance-associated mutations in Plasmodium falciparum circulating in 2017–2018, Bo, Sierra Leone
Source: Front Microbiol. 2022 Dec 2;13:1059695. doi: 10.3389/fmicb.2022.1059695 (PMC9755742; doi:10.3389/fmicb.2022.1059695)
Supplement: Supplementary file 1 [file Data_Sheet_1.docx]

**Prevalence of malaria resistance mutations in *Plasmodium falciparum* circulating in 2017-2018, Bo, Sierra Leone**

**Supplementary Materials**

**Supplementary Methods**

**Multiplexed PCR amplifications – main method based on Nag et al. [1]**

The amplifications were conducted using FastStart Taq DNA polymerase system (MilliporeSigma, Burlington, MA, USA) according to manufacturer’s recommendations. The PCR reactions were set up in 20 µL total volume and included 2U of the enzyme, 2 mM MgCl_2_, 200 nM of each primer (from 2 to 8 primers depending on the reaction) and 200 µM of dNTPs and 2 µL of the template DNA. The amplifications were conducted using Quant Studio 5 thermal cycler (Thermo Fisher, Grand Island, NY) using the following thermal cycling program: 4 min at 95 °C, 32 cycles of 30 sec denaturation at 95 °C, 1 min annealing at 57 °C, 2 min extension at 72 °C followed by 7 min final extension at 72 °C.

**PCR amplifications optimized for individual amplicons (this work)**

The second round of amplifications was conducted after obtaining the results of the first sequencing run with the purpose of filling in the gaps in the sequence data set therefore only the samples for which no good quality sequence was obtained were included for each amplicon. The PCR mix composition for these amplifications was identical as for the first round of amplifications. Based on the results of conducted PCR amplification optimization the PCR cycling conditions and annealing temperatures were adjusted depending on the amplicon. For *pfcrt* and *pfmdr1* amplifications the annealing temperature of 62 °C and 35 amplification cycles were used while for *pfdhps*, *pfkelch13* and mitochondrial fragments, annealing temperature of 60 °C and 38 amplification cycles were used. There was no need to perform additional amplification of *pfdhfr* fragments.

**Indexing reactions**

Each indexing reaction used unique forward and reverse indexing primers, the forward indexing primers #1 through #7 and #18 through #20 and reverse indexing primers #1 through #10 and #18 through #20 were used see Supplementary Table 2. The indexing amplifications were conducted using FastStart Taq DNA polymerase system (MilliporeSigma) using the same mastermix composition as used for amplicon generation and using 2 µL of pooled amplicons as a template. The thermal cycling program was also the same with the exception of the annealing temperature was set for 60 °C and included 32 amplification cycles.

**Supplementary Discussion of sequencing success rates**

A version of a previously described high-throughput method using multiplex PCR, custom barcoding for sample multiplexing and Illumina MiSeq high-throughput sequencing was used in this study [1]. The authors of the original method initially reported extremely high sequencing success rates (above 94% of all targeted polymorphic sites in all amplified fragments were successfully sequenced) however, in their subsequent study from the same group the authors were not able to match these results mainly due to low rates of PCR success (from 13% to 87%) for many amplicons despite switching from multiplex to single PCR reactions, using nested PCR and employing other optimization steps [2]. In the study described here two rounds of sequencing were performed - the first one using the original method which resulted in very low success rates for certain amplicons (e.g., 0% for *pfkelch13* amplicon 1 and 16% for *pfcrt*) and the second one, using optimized single-plex PCR reactions, with the purpose of filling the gaps in the dataset resulting in significantly improved success rates, see Supplementary Table 3. The reasons of low sequencing success for some amplicons are not clear. Biased amplification of fragments in multiplex-PCR reactions may be one reason, however in some cases, such as *pfcrt*, where single-plex amplification was used the bias during sequencing is a more likely cause. The differences of overall success rates presented here and the ones published by authors of the original method may also be results of using different starting materials (whole blood vs. blood stored on filter paper), different DNA extraction methods and/or using PCR reagents from different manufacturers.

**Data availability**

Raw sequence reads were deposited into a publicly available database at the National Center for Biotechnology Information Sequence Read Archive (NCBI SRA) under BioProject ID: PRJNA904762

http://www.ncbi.nlm.nih.gov/bioproject/904762

**Supplementary Table 1. Sequences of primers used for amplification of gene fragments containing drug resistance SNPs.**

| Name | Target | Sequence | Multiplex | Ref. |
| --- | --- | --- | --- | --- |
| Pfcrt.2-F | *Pfcrt* | TCGTCGGCAGCGTCAGATGTGTATAAGAGACAG**TGGCTCACGTTTAGGTGGAGGTTCTTG** | n/a | [1] |
| Pfcrt.2-R |  | GTCTCGTGGGCTCGGAGATGTGTATAAGAGACAG**ACTGAACAGGCATCTAACATGGATATAGC** |  |  |
| Pfmdr1.1a-F | *Pfmdr* | TCGTCGGCAGCGTCAGATGTGTATAAGAGACAG**TGAACAAAAAGAGTACCGCTGA** | #1 | this work |
| Pfmdr1.1a-R |  | GTCTCGTGGGCTCGGAGATGTGTATAAGAGACAG**AACGGAAAAACGCAAGTAATACA** |  |  |
| Pfmdr1.7-F |  | TCGTCGGCAGCGTCAGATGTGTATAAGAGACAG**TTTGTCCAATTGTTGCAGCTGTATTAACTTT** | #4 | [1] |
| Pfmdr1.7-R |  | GTCTCGTGGGCTCGGAGATGTGTATAAGAGACAG**TGCATTTTCTGAATCTCCTTTTAAGGACATT** |  |  |
| Pfmdr1.8-F |  | TCGTCGGCAGCGTCAGATGTGTATAAGAGACAG**GGTAAAGTTGATATTAAAGATGTAAATTTCC** | #3 |  |
| Pfmdr1.8-R |  | GTCTCGTGGGCTCGGAGATGTGTATAAGAGACAG**TGGTCCAACATTTGTATCATATTTATTTGG** |  |  |
| Pfdhfr.1-F | *Pfdhfr* | TCGTCGGCAGCGTCAGATGTGTATAAGAGACAG**ATGATGGAACAAGTCTGCGACGTTTTCGA** | #2 |  |
| Pfdhfr.1-R |  | GTCTCGTGGGCTCGGAGATGTGTATAAGAGACAG**CTAAAAATTCTTGATAAACAACGGAACCTCC** |  |  |
| Pfdhps.3-F | *Pfdhps* | TCGTCGGCAGCGTCAGATGTGTATAAGAGACAG**ACCATCAGATGTTTATATAACAAATATGTG** | #3 |  |
| Pfdhps.3-R |  | GTCTCGTGGGCTCGGAGATGTGTATAAGAGACAG**CTGGATTATTTGTACAAGCACTAATATCA** |  |  |
| Pfdhps.4-F |  | TCGTCGGCAGCGTCAGATGTGTATAAGAGACAG**AGAATGTGTTGATAATGATTTAGTTGATAT** | #4 |  |
| Pfdhps.4-R |  | GTCTCGTGGGCTCGGAGATGTGTATAAGAGACAG**GATATAAAAGTTGATCCTTGTCTTTCCT** |  |  |
| PfK13.1-F | *Pfkelch13* | TCGTCGGCAGCGTCAGATGTGTATAAGAGACAG**ATGGAAGGAGAAAAAGTAAAAACAAAAGC** | #1 |  |
| PfK13.1-R |  | GTCTCGTGGGCTCGGAGATGTGTATAAGAGACAG**GTTGGTATTCATAATTGATGGAGAATTC** |  |  |
| PfK13.2-F |  | TCGTCGGCAGCGTCAGATGTGTATAAGAGACAG**CTGACAGCAAATAATATAACTAATAATCT** | #3 |  |
| PfK13.2-R |  | GTCTCGTGGGCTCGGAGATGTGTATAAGAGACAG**TCTTCATCAAATCGTTTCCTATGTT** |  |  |
| PfK13.3-F |  | TCGTCGGCAGCGTCAGATGTGTATAAGAGACAG**GAGTACGATTGTACAAAGAATTAGAAAACCG** | #1 |  |
| PfK13.3-R |  | GTCTCGTGGGCTCGGAGATGTGTATAAGAGACAG**ATAACTCACTATCCCTATCTAAGAATATTC** |  |  |
| PfK13.4-F |  | TCGTCGGCAGCGTCAGATGTGTATAAGAGACAG**TAAGTGGAAGACATCATGTAACCAGAGA** | #2 |  |
| PfK13.4-R |  | GTCTCGTGGGCTCGGAGATGTGTATAAGAGACAG**CTTCTACATTCGGTATAATAGAAGAGCC** |  |  |
| PfK13.5-F |  | TCGTCGGCAGCGTCAGATGTGTATAAGAGACAG**ATGATGGCTCTTCTATTATACCGAATG** | #1 |  |
| PfK13.5-R |  | GTCTCGTGGGCTCGGAGATGTGTATAAGAGACAG**GCTATTAAAACGGAGTGACCAAATCTG** |  |  |
| Mito.1-F | *P. falciparum* mitochondrial genome | TCGTCGGCAGCGTCAGATGTGTATAAGAGACAG**CTTCCCTTCTCGCCATTTGATAGCGG** | #5 |  |
| Mito.1-R |  | GTCTCGTGGGCTCGGAGATGTGTATAAGAGACAG**GAAAAAGGAATGAGTTTTGAAATCTCTAGTA** |  |  |
| Mito.2-F |  | TCGTCGGCAGCGTCAGATGTGTATAAGAGACAG**GATATGATAAATGTAAATACTCTGTAGTTTG** | n/a |  |
| Mito.2-R |  | GTCTCGTGGGCTCGGAGATGTGTATAAGAGACAG**CCTGCATTAACATCATTATATGGTACATC** |  |  |
| Mito.3-F |  | TCGTCGGCAGCGTCAGATGTGTATAAGAGACAGCAG**TAATACTACGTACTGAATTATATTCTTC** | #5 |  |
| Mito.3-R |  | GTCTCGTGGGCTCGGAGATGTGTATAAGAGACAG**GAAGACATAATACTAGCGACTCCAGATAC** |  |  |

**Supplementary Table 2. Sequences of indexing primers used in this study.**

| Name | Sequence | Ref. |
| --- | --- | --- |
| Index_1_F | AATGATACGGCGACCACCGAGATCTACACaaaagggaTCGTCGGCAGCGT | [1] |
| Index_2_F | AATGATACGGCGACCACCGAGATCTACACaaacagccTCGTCGGCAGCGT |  |
| Index_3_F | AATGATACGGCGACCACCGAGATCTACACaaactcgcTCGTCGGCAGCGT |  |
| Index_4_F | AATGATACGGCGACCACCGAGATCTACACaaagacacTCGTCGGCAGCGT |  |
| Index_5_F | AATGATACGGCGACCACCGAGATCTACACtttcctacTCGTCGGCAGCGT |  |
| Index_6_F | AATGATACGGCGACCACCGAGATCTACACtggttacaTCGTCGGCAGCGT |  |
| Index_7_F | AATGATACGGCGACCACCGAGATCTACACtgcaccatTCGTCGGCAGCGT |  |
| Index_18_F | AATGATACGGCGACCACCGAGATCTACACcacaggtgTCGTCGGCAGCGT |  |
| Index_19_F | AATGATACGGCGACCACCGAGATCTACACcagtactaTCGTCGGCAGCGT |  |
| Index_20_F | AATGATACGGCGACCACCGAGATCTACACcatgcttgTCGTCGGCAGCGT |  |
| Index_1_R | CAAGCAGAAGACGGCATACGAGATtcccttttGTCTCGTGGGCTCGGAGA |  |
| Index_2_R | CAAGCAGAAGACGGCATACGAGATggctgtttGTCTCGTGGGCTCGGAGA |  |
| Index_3_R | CAAGCAGAAGACGGCATACGAGATgcgagtttGTCTCGTGGGCTCGGAGA |  |
| Index_4_R | CAAGCAGAAGACGGCATACGAGATgtgtctttGTCTCGTGGGCTCGGAGA |  |
| Index_5_R | CAAGCAGAAGACGGCATACGAGATgtaggaaaGTCTCGTGGGCTCGGAGA |  |
| Index_6_R | CAAGCAGAAGACGGCATACGAGATtgtaaccaGTCTCGTGGGCTCGGAGA |  |
| Index_7_R | CAAGCAGAAGACGGCATACGAGATatggtgcaGTCTCGTGGGCTCGGAGA |  |
| Index_8_R | CAAGCAGAAGACGGCATACGAGATaactcagaGTCTCGTGGGCTCGGAGA |  |
| Index_9_R | CAAGCAGAAGACGGCATACGAGATccatgtgtGTCTCGTGGGCTCGGAGA |  |
| Index_10_R | CAAGCAGAAGACGGCATACGAGATgaagacgtGTCTCGTGGGCTCGGAGA |  |
| Index_18_R | CAAGCAGAAGACGGCATACGAGATcacctgtgGTCTCGTGGGCTCGGAGA |  |
| Index_19_R | CAAGCAGAAGACGGCATACGAGATtagtactgGTCTCGTGGGCTCGGAGA |  |
| Index_20_R | CAAGCAGAAGACGGCATACGAGATcaagcatgGTCTCGTGGGCTCGGAGA |  |

**Supplementary Table 3. Sequencing success rates.**

| Amplicon | Sequencing success rates (%)^1^ | |
| --- | --- | --- |
|  | First sequencing run | Combined sequencing runs |
| *Pfcrt* | 16 | 65 |
| *Pfmdr1*-1 | 95 | 97 |
| *Pfmdr1*-7 | 29 | 83 |
| *Pfmdr1*-8 | 81 | 81 |
| *Pfdhfr* | 100 | 100 |
| *Pfdhps*-3 | 57 | 94 |
| *Pfdhps*-4 | 35 | 90 |
| *Pfkelch13*-1 | 0 | 95 |
| *Pfkelch13*-2 | 51 | 84 |
| *Pfkelch13*-3 | 100 | 100 |
| *Pfkelch13*-4 | 91 | 96 |
| *Pfkelch13*-5 | 100 | 100 |
| Mito-1 | 85 | 97 |
| Mito-2 | 100 | 100 |
| Mito-3 | 100 | 100 |

^1^The success rates reflect the proportion of sequences of adequate quality to determine the bases at SNP positions out of all included samples. In case of presence of multiple SNPs with different success rates in a single amplicon, the lowest value was shown.

References

1. Nag S, Dalgaard MD, Kofoed P-E, Ursing J, Crespo M, Andersen LOB, Aarestrup FM, Lund O, Alifrangis M: **High throughput resistance profiling of *Plasmodium falciparum* infections based on custom dual indexing and Illumina next generation sequencing-technology.** *Scientific Reports* 2017, **7:**2398.

2. Nag S, Ursing J, Rodrigues A, Crespo M, Krogsgaard C, Lund O, Aarestrup FM, Alifrangis M, Kofoed PE: **Proof of concept: used malaria rapid diagnostic tests applied for parallel sequencing for surveillance of molecular markers of anti-malarial resistance in Bissau, Guinea-Bissau during 2014-2017.** *Malar J* 2019, **18:**252.
